# Access to infertility care services towards Universal Health Coverage is a right and not an option

**DOI:** 10.1186/s12913-022-08456-7

**Published:** 2022-08-25

**Authors:** Rachid Bezad, Sanae El Omrani, Amal Benbella, Bouchra Assarag

**Affiliations:** 1 Obstetrics and Gynecology, Assisted Reproductive Technology Unit of the Reproductive Health Center, University Hospital Ibn Sina, Rabat, Morocco; 2 Faculty of Medicine and Pharmacy, University Mohamed V, Rabat, Morocco; 3 Maternal and Neonatal Health, University Ibn Tofail, Kenitra, Morocco; 4 The Association Together for Reproductive Health (ESR), Rabat, Morocco; 5 The Association Together for Reproductive Health (ESR), National School of Public Health, Rabat, Morocco

**Keywords:** Infertility, SRH, Universal health coverage, Priority settings, Right

## Abstract

**Background:**

In Morocco, the national health plan 2025 was developed to promote Sexual and Reproductive Health (SRH) services for all. The principal aim was to achieve the Universal Health Coverage of SRH by 2030. For many years, health authorities’ efforts had focused on reducing maternal mortality through a widespread access to antenatal and obstetric care and family planning services. This has resulted in a significant gap between the availability of SRH components, namely obstetric and family planning care, and access to infertility services including Assisted Reproductive Technology (ART). The objective of this study is to answer two important questions. First, why some SRH programs and services are given priority by international and national political leaders while infertility care receives little attention; second, what are the factors that influence this prioritization?

**Methods:**

We used Shiffman and Smith’s framework composed of four elements: the strength of the actors involved in the initiative, the power of the ideas they use to represent the health problem, the nature of the political contexts in which they operate and the characteristics of the services. We added a fifth element to the framework, the outcome. We applied this framework to the case of infertility services in Morocco. We conducted a desk review and interviews with actors involved in SRH and infertility care advocates as well as with decision makers involved in implementing Universal Health Coverage (UHC).

**Results:**

Our results showed that despite the efforts made by the advocates of infertility care; the enactment in 2019 of a law regulating infertility care services; and the presence of two Assisted Reproductive Technology Units in the public sector, infertility services remain at an early stage of development hampered by multiple challenges. Among others, a lack of political entrepreneurs to ensure a strong leadership; the political windows were often missed; community members lacked consensus on a coherent public positioning of the problem, and advocates' perception and power of the idea lacked evidence and precise indicators of the problem.

**Conclusion:**

To ensure the convergence and alignment of all stakeholders, it is recommended to translate the regulation of infertility into measurable activities with defined human and financial resources, equitable fertility health coverage, and quality fertility care to respond to women and infertile couples’ needs, rights and dignity.

## Introduction

Universal access to a comprehensive range of Sexual and Reproductive Health (SRH) services is fundamental to achieving Universal Health Coverage (UHC) as suggested by the Sustainable Development Goal 3.^1^ Being committed to universal sexual reproductive health coverage by 2030, Morocco is still exploring the existing resources and channels to define the SRH essential health benefit package, which needs to include fertility care services.

Since 1970, Reproductive and Maternal Health (RMH) in Morocco evolved through several stages in Morocco. Amongst the main evolving phases, the 1980s were focused on establishing safe motherhood and family planning units at primary health care facilities based on Alma Ata Recommendations. The 1990s targeted the rights of women and couples in accessing and benefiting from comprehensive reproductive and maternal health services based on the ICPD recommendations [1]. During the period between 2000 and 2007, there was an upgrading of family planning and safe motherhood services with, on one hand, the institutionalization of national health policies, programs and service deliveries; on the other hand, the security of modern methods of family planning and commodities. In 2008, the Ministry of Health (MOH) developed the first national health action plan (2008–2012) with the involvement of all related departments at the Ministry of Health and stakeholders involved in population’s health and wellbeing through strengthening health coverage [2]. The national plan stressed the importance of adopting multisectoral and integrated approaches in establishing RMH packages for primary and secondary health care service delivery [3].

In the meantime, the first national strategy on reproductive and maternal health 2011–2020 was developed. It included the key components of sexual and reproductive health along with the continuum of care. It also engaged all the concerned MOH units and departments to adopt a joint plan for a better-integrated RMH service delivery [4].

Based on the key achievements of the national action plan 2008–2012, an acceleration plan was developed during 2012–2016. Its purpose was to reinforce SRH services and target evidence based, cost effective and high impact reproductive and maternal health interventions to accelerate the reduction of maternal and new-born mortality, by ensuring equitable access and coverage of SRH along the continuum of care towards UHC [5, 6].

To strengthen the SRH package, further SRH interventions were added and included such as the prevention and management of sexually transmitted diseases including HIV/AIDS; SRH services for adolescents, gender-based violence care; and breast and cervical cancers screening and management. The revised SRH package highlights the importance of the integration and comprehensiveness of service delivery at the Primary Health Care (PHC) level [4].

During the last decade, Morocco has put maternal health as a national priority with the involvement of all partners and stakeholders. These health policies and interventions have led to a reduction of the maternal mortality ratio by more than 78% in twenty-six years from 332 maternal deaths per 100,000 live births in 1992 to 72.6 deaths per 100 000 live births in 2018. In addition, contraceptive prevalence among married women aged 15–49 increased significantly from 42% in 1992 to 70.8% in 2018 [7]. In fact, the unmet need for family planning has decreased from 19.7% in 1992 to 11.3% in 2018 [7]. Moreover, the antenatal health care coverage of four visits and plus reached 88.5%. Finally, the percentage of births taking place in health facilities was 86.6% in 2018 [7].

Targeting a universal SRH coverage by 2030, the national health plan 2025 was developed by engaging the concerned SRH departments, related actors and partners to promote SRH for all [8]. Linkages between different SRH components were highlighted as well as the convergence towards an integrated SRH package delivery with accessibility, availability and affordability of the SRH life-saving and essential interventions while improving the SRH coverage, ensuring an integrated benefit package delivered at PHC and hospital levels. However, Morocco is still facing some challenges regarding disparities between urban and rural areas; between high and low educated population; and between high and low socioeconomic status groups [1].

Moreover, some SRH components, such as infertility prevention and management services, although they were mentioned in the national RH strategy 2010–2019, yet their translation into actions and interventions at health facilities was not well accomplished. Indeed, infertility law 47–14 governing medically assisted procreation (MAP) was recently promulgated [9]. However, access to these services for women and couples remain limited and not fulfilling the right of women and the values of practices [7].

According to the WHO, infertility is defined as a disease due to an impairment of function reflected through lack of pregnancy after 12 months of unprotected intercourse [10]. Infertility is estimated to affect as many as 186 million people worldwide, it is higher in low-income countries [11] according to a national survey conducted in 2015 [12].

Infertility remains a public health issue in Morocco, with an estimated prevalence of approximately 12%; and the average duration of the first medical visit seeking care at a health facility is estimated to be five years [13]. The delay in seeking care is due to the limited access, availability and affordability of ART treatment. Furthermore, infertility prevention and management services are not integrated within the existing SRH Package, and are not covered by the available insurance schemes. Due to the latter, many infertile couples incur catastrophic expenditures to fund ART, or engage in cross-border reproductive care (CBRC) to seek low-cost IVF services outside Morocco [11, 14, 15]. In Morocco, despite a growing awareness of infertility causes and treatment possibilities, infertility is still linked to women who bear the consequences, suffering from social stigmatisation and marital problems leading to divorce or polygamy. The consequence of such social and mental pressure led to the occurrence of various psychological disorders in infertile Moroccan women, with 55% of the women having depression and 45.6% having mild to severe anxiety [9].

Morocco has established three systems of health insurance in 2002 including (i) Mandatory Health Insurance (AMO), (ii) Medical Assistance Scheme for the Economically Underprivileged (RAMED) and (iii) Medical Insurance for the Self-Employed; yet up to date infertility prevention and management services are not included within the health insurance schemes which is a hindrance to the progress towards UHC [8].

The role of different SRH actors is crucial in the prioritization of infertility care among the SRH benefit package. Therefore, ensuring an equitable package by considering infertility care as the couple’s right and not just an option, should be a priority of the Moroccan health system [15].

To understand the ‘why’ and ‘how’ of the prioritization of SRH over the last two decades in Morocco, this study focused on the policy considerations that influence SRH agenda priority setting, and decision making for the financial and budgetary allocations for infertility care services implementation.

## Methods

### Approach

To conduct this study, we adopted Shiffman and Smith framework to assess the health policy priority setting for SRH package including infertility care services [16]. Using this framework, we explored four categories: the strength of the actors involved in the initiative, the power of the ideas they use to portray the issue, the nature of the political contexts in which they operate, and characteristics of the issue itself [17]. We added a fifth additional element to the framework, the outcome. This element helped to identify if the issue under discussion was taken seriously and prioritized by decision-makers, and if it was adopted by higher authorities along with resources available for implementation [17] (Table 1).

**Table 1:** Framework for assessing what factors affect national agenda setting, adapted from the Shiffman and Smith framework

Elements	Description	Factors shaping policy priorities
Actor power	The strength of the individuals and networks concerned with the issue	1. Policy community cohesion: the degree of coalescence in the network involved with the issue 2. Leadership: the presence of individuals capable of uniting the policy community, acknowledged as strong champions 3. Guiding institutions: effectiveness of organizations or co-ordinating mechanisms 4. Civil society mobilization: the extent to which grassroots organizations are mobilized to support action
Ideas	The ways in which those involved with the issue understand and portray it	1. Internal frame: the degree to which the policy community agrees on the definition of, causes and solutions to, the problem 2. External frame: public portrayals of the issue in ways that resonate with external actors, especially the political leaders who control resources
Context	The environment in which actors operate	1. Policy windows: political moments when conditions align favorably for an issue, presenting opportunities for advocates to influence decision makers 2. Global governance structure: the degree to which norms and institutions operating in a sector provide a platform for effective collective action
Issue characteristics	Features of the problem	1. Credible indicators: clear measures that show the severity of the problem, which can be used to monitor progress 2. Severity: the size of the burden relative to others 3. Effective interventions: the extent to which proposed means of addressing the problem is explained, cost effective, backed by scientific evidence, simple to implement and inexpensive
Outcome	Helps to judge how strongly the issue is on the agenda	Decision in the policy process that leads to change: e.g. allocation of resources should be broken down into financial, technical and human resources

### Study design, population and sampling

This is an exploratory qualitative case study conducted in Morocco. It is based on a desk review to map and analyze documents regarding infertility care integration within Sexual and Reproductive Health Benefits Package towards Universal Health Coverage over the last two decades in Morocco. The study was conducted in Rabat the capital of Morocco where most of health policymakers and program managers of SRH are based as well as stakeholders, concerned partners, private sectors and NGOs. This study was carried out between November 2019 and May 2020.

The population surveyed included policymakers and actors involved in health priority-setting process in order to identify the fundamental values related to provisions for population coverage of UHC in Morocco, especially the integration of infertility care services on the SRH benefit package (see Table 2). The actors selected are those who were involved in the process of developing the national reproductive health strategy in Morocco, which involves the organization of workshops to debate and reach consensus on national priorities and the development and validation of the 10-year national reproductive health strategy.

**Table 2:** Interviews of key informants and where they worked

Health system level	Total
National health insurance	**3**
Ministry of Health (MOH)	**11**
Development partners	**3**
NGOs	**3**
Service delivery/technical expert	**4**
Health workers	**8**
Community/women	**13**

National stakeholders were stratified into three groups:

- National government stakeholders responsible for defining national reproductive health programs and the national health financing policy in the country;
- Related partners and stakeholders who provide both financial and technical support on health systems strategies, including the private sector;
- Civil societies and NGOs. In addition, we added key informants from women/couples and health workers.

Regarding the participant selection, we considered that including all stakeholders was useful to draw up a global view and gain a historical perspective on the reform processes and the involvement of different actors on SRH care services within universal health coverage in Morocco, with a special interest to the infertility care services which still appears to be problematic. In fact, we included in our stratified sample, the health care providers at different levels (operational, provincial and regional), as well as the private sector; decision-makers and actors from other departments involved in the management of financial and support mechanisms for SRH programs and universal health coverage in Morocco. We also included the vulnerable groups (infertile couples) whose authorial voice added richness to the analysis. Indeed, recruitment of the infertile couples was facilitated through NGOs after their informed consent. A specific consent form was presented and explained to each participant and interviewee with the insurance that their anonymity will be preserved.

### Data collection

We used two main methods for data collection: a desk review and key informant interviews.

A data extraction sheet was used for the review of scientific articles, reports, MOH reports and national documents, related partners reports and documents, and relevant independent research report and papers. The desk review was limited to English and French documents which were published between January 2000 and December 2019, and without any methodological restrictions.

Semi-structured interview guides adapted to each category of our sample, presented in Table 2, were used to discuss the following questions:

1. Who were the key actors involved in infertility care priority-setting within UHC plan at different levels of the Ministry of Health during the reform process?
2. How important is it for infertility care services actors to be involved in priority-setting processes?
3. How far are infertility care services priorities reflected in SRH benefit package?
4. Are the priority-setting processes sensitive to the needs and national priorities of SRH services?
5. What policy decision was being taken by policymakers to successfully advance access to infertility care services and related rights in Morocco?

A focus group guide was elaborated based on the preliminary results of the individual interviews in order to explore in depth some issues.

The interviews and focus group discussions were recorded after the informed consent of the participants.

### Analysis

The guiding framework for our analysis was the various components of the adapted framework from Shiffman and Smith (2007). The analysis of 50 documents provided an effective and valuable tool for researchers who were undertaking comparative studies of policy implementation [18].

The sheet, used for the analysis of the selected documents, was structured and guided by the key items of the study framework. In addition, the documentary analysis provided supplementary data that we used to contextualize and clarify the second method of data collection used for this case study (interviews and group discussion).

The interviews and focus groups were fully transcribed and analyzed manually, by the health sociologist, using the "content analysis" approach; the process of analysis reduced the volume of text collected, identified group categories together and sought some understanding of it [19]. In some way, we attempted to “stay true” to the text and to achieve trustworthiness [20].

The analysis included the following steps:

1. Transcription of interviews and focus group discussions, highlighting a global understanding of the interviewees' points of view.
2. Classification of the interview and focus group data according to the themes and the sub-themes identified. Items and categories were identified based on research framework.
3. Data analysis of the interview and the focus group was supported by the sociologist's expertise in this area.

### Ethical considerations

The study was approved by the Ethics Committee of Biomedical Research of the University Mohammed V of Rabat, under reference: 17/20 on January 17^th^ 2020; and WHO Ethics Review Committee.

Individual written consent was sought from all study participants. The participants were adequately informed of the purpose of the study, their willingness to participate in the study without any coercion, the benefits and adverse effects of participating as well as the preservation of their confidentiality if they participated to the study. They were also informed that they are free to withdraw from the study at any stage. Participation in this study did not involve any risk to the participants.

## Results and discussion

Forty-five participants were interviewed during this study (Table 3). The interviews took place between November 2019 and February 2020 with additional information collected in May 2020. The different outputs of our results followed the study framework. Below we highlighted through quotes, the barriers and facilitators of generating political priority for infertility services within the UHC.

**Table 3:** Characteristics of participants

Code	ID	Number	Institution
DP1	Female	4	Government
DP2	Female		Government
DP3	Female		Government
DP4	Male		Government
DC1	Female	1	Government
DELM1	Female	2	Government
DELM2	Female		Government
RAMED1	Male	1	Government
DPRF1	Male	2	Government
DPRF2	Female		Government
GynecoPUB	Male	2	Providers
SFPub	Female		Providers
GynecoPR1	Female	2	Providers
GynecoPR2	Male		Providers
ANAM1	Male	1	Government
CNOPS1	Male	1	Government
CNSS1	Male	1	Government
NU1	Female	2	International Development Agency
NU2	Male		International Development Agency
NU3	Female	1	International Development Agency
ONGR1	Female	1	NGO
ONGU2	Female	2	NGO
INFCSR1	Female	1	Providers
MEDGENU1	Male	2	Providers
SFU1	Female		Providers
SFPUBR1	Female	1	Providers
MIG1	Female Male	2	Community
PVVIH1	Female	1	Community
PVVIH2	Male	1	Community
AS1	Female	1	Community
JSCOL1	Female	4	Community
JSCOL2	Female		Community
JNSCOL1	Male		Community
JNSCOL2	Male		Community
Part1	Female Female	2	Community
WMAPA1	Female	2	Community
WMAPA2	Female		Community
DIRORAN1	Male	1	Technique expert and Providers
FG1	3 Female 1 Male	4	Government + NGO + Community + health worker
Total		45	

### Actor power in shaping SRH policy priorities within the UHC

Priority-setting is a central part of building efficient, responsive and resilient healthcare systems [20]. Actor power in the Shiffman and Smith (2007) framework is explained as ‘the strength of the individuals and organizations concerned with the issue. Actors influence the policy making process through their knowledge, experiences, beliefs and power [21].

In Morocco, there is a wide range of actors in the SRH landscape ranging from government, researchers and national technical experts, private sector partners, civil society and NGOs. While there was congruence on the progress made on attaining UHC, several stakeholders have expressed that national efforts by health authorities have focused on reducing maternal mortality and morbidity which has been a national priority through a widespread access to antenatal and obstetric care and family planning services. This has led to a large gap within which SRH services have been implemented. Indeed, obstetric care and family planning services are more developed than infertility care services, including assisted reproductive technology:

“……couples with infertility problems suffer in silence, and face several obstacles; cultural, social, economic and geographic. All those factors make the access to health services difficult…. this is why we have organized, in collaboration with scientific experts, meetings where we had invited health policymakers and the National Agency of Health Insurance to help the voice of these couples, needing health services, be heard and their human rights and their dignity respected” (NGO).

Most interviewees expressed the main role of the private sector as a pioneer in the introduction of infertility care services in Morocco:

“…….no one can deny that the private sector was the first to set up and develop infertility services in Morocco …… but the cost is very high and access to these services was elective and economically discriminatory” (national technical expert).

Therefore, health policymakers respondents acknowledged that national health strategies have always been focused on reducing total fertility rates while infertility care has received little attention. To date, there are only two public centers for infertility care services providing ART; the first based in Rabat and the second located in Marrakech:

“…….thanks to a technical expert team, the first center for medically assisted procreation has been established in Morocco in Rabat as part of a project by the University Hospital Center and a specialized center for infertility management in Belgium in order to reduce the cost of infertility services in Morocco and enable the economically disadvantaged couples to access infertility services at a public health facility” (technical expert).

Another point of discussion was what stakeholders viewed as the complexity of financial decision-making amongst those with limited disposable income: in their view, several stakeholders accused the national government of lacking a holistic approach towards access to infertility care services, and missing health coverage and financial protection [22]. A coalition was formed to draw attention to this public health matter:

“……a memorandum has been sent in the form of a claim book to the Head of Government and the Minister of Health to explain the infertility issue. The drafting of this memorandum is the result of a national solidarity campaign to support the infertile couples. This petition was signed by couples, their family members, journalists, doctors, pharmacists, health industry actors, and citizens’ sensitive to this issue, but also NGOs, nurses, students, lawyers….”. (NGO).

However, in the policy-making space, differential power existed; United Nations agencies for example play an important role on both SRH and UHC issues, depending on their international norms and guidelines. Priority-setting process theoretically allows a diverse range of healthcare stakeholders to articulate their preferred values and agendas in order to achieve consensus on the direction for a country’s health agenda [23]. However, based on the interviews it was noted that the key SRH partners expressed divers contributions in the process of infertility care services prioritization and its inclusion within the Health Benefit Package (HBP). A UN Agency respondent mentioned the importance of SRH as a key component of UHC, yet they acknowledged the inequitable coverage.

“…..we have supported the Ministry of Health for years in the various components of reproductive and sexual health and UHC; to provide a comprehensive and integrated RH package to the entire population with financial protection….But inequity and disparity to access health services and the poor quality of care remain a national challenge toward UHC, we provide the technical and financial support to the government to achieve the national RH priorities within UHC.” (UN Agency).

UN partners recognized the infertility component as a key intervention for SRH package based on human rights approach and gender equality, as expressed through this verbatim;

“….focus in achieving universal access to Sexual and Reproductive Health (SRH), the promotion of reproductive rights, monitoring population dynamics, adopting the human rights approach and gender equality, we have supported the Ministry of health in the field of infertility to develop a national infertility action plan…..” (UN Agency).

Further, it was noted that prioritization of infertility care services among SRH package depends on the government national health priorities, as well as the convergence with available resources. Following a joined advocacy from partners and policymakers, Morocco has established a Law on the medically assisted procreation that was adopted in 2019, in response to the population needs and to ensure the respect of their rights and dignity. This law recognized for the first time that infertility is considered as a disease which requires prevention and management measures within HBP.

The establishment of the infertility law was a result of joined efforts deployed by policymakers, program managers, UN agencies and NGOs. However, according to interviews, more work has to be conducted in insuring coherence of the policy community of infertility stakeholders as well as strengthening the leadership of the MOH in fulfilling the requirements for integrating infertility care within SRH benefit package.

### Ideas impacting on SRH interventions priority setting

Priority setting of health interventions to support low- and middle-income countries (LMICs) in their strive for universal health coverage (UHC) are mainly geared towards the development of more cost-effectiveness information, and this evidence does not sufficiently support countries to make optimal choices [24]. Indeed, a majority of respondents felt that accessible infertility treatment could only be successfully introduced if socio-cultural, economic prerequisites are fulfilled, and governments can be persuaded to support their introduction.

Key informants representing the different stakeholder groups viewed that infertility has not been recognized by policy makers as a disease but only as a desire, and the issue has not been considered a public health problem:

“…we are a forgotten population; we are facing several problems without any support… we can't find the information we need. For many years I was searching for doctors across the country, all what I gained from work was dedicated to medical fees for 12 years and I still hope to have a child because I feel as an incomplete woman and the society makes me feel like this” (civil society representative).

Respondents across community and technical expert networks acknowledged that the Infertility care is probably the most neglected and underestimated health care issue, as mentioned by the head of NGO:

“……stakeholders must understand that infertility is a public health problem and the consequences of involuntary childlessness are much more dramatic than policymakers can imagine, particularly for women. Negative psychosocial consequences are often severe and childless women are frequently stigmatized, isolated, and neglected by the entire family and even the community….and they need affordable services equally to the other vulnerable populations…..” (Civil society representative).

However, health policymakers need indicators integrated within the national health information system and socio-anthropologic and demographic studies to make decisions based on multiple criteria beyond cost-effectiveness:

“…..The current information system does not cover the activity of the private sector, whereas its activities are very important in the area of ​​SRH, particularly infertility, abortion care services, cervical cancer screening, etc. Collecting information from all of these activities will improve the possibility of estimating the costs and to determine the scope of health problems…”. (MOH official).

A stakeholder raised the possibility that global goals and donor priorities may influence the government's SRH strategy, as the prevention and management of infertility has recently gained prominence in international priorities [25]:

“…..In 2018, we attended an international assembly on infertility, it is starting to take its place on the priorities of the global agenda. But, still infertility don’t really have the appropriate position in SRH because it isn’t like Maternal and Child Mortality, which is a concern because of the Millennium Development Goals and currently with the Sustainable Development Goals targets…”. (technical expert).

Decision-making on sexuality and reproductive health should be more from a right-based approach and gender inequalities, to ensure equity in health care access, provide adequate and quality healthcare services to reach population needs and decrease out-of-pocket (OOP) health expenditures by households.

### Political context provides opportunities to seize policy windows

When probed about Moroccan political environment, we observed a consensus in the views expressed by the respondents. The health policy reforms were consolidated by the adoption of the 2011 Constitution, which reinforces the fundamental rights of citizens and stresses the mobilization of all available means to facilitate equal access for all the population to the conditions allowing them to enjoy rights of access to health care. In addition, the improvement and extension of universal health coverage is one of the pillars of the human and social development advocated by His Majesty King Mohammed VI. For this purpose, two schemes of basic health coverage were created in 2002, as well as the advanced regionalization policy:

“There is the king's speech; there is a will from the highest political authority in the country. Then the commitment on the government declaration which includes UHC as an axis of government policy”. (MOH official).

Respondents acknowledged that the approach adopted by the Ministry of Health to develop the health national strategic plan 2020–2025 was very participative and allowed actors and partners involved to participate in the process. Indeed, for the first time, the infertility care services are integrated within the national strategic health plan:

“In Morocco, currently, the 2025 national health plan includes the reproductive health priorities to achieve the SDGs, this plan, prepared by the Ministry of Health and concerned partners, includes the reproductive health progress achievements and the development of new programs, like infertility care services…. However, the infertility care services action plan was planned without allocation of financial resources. Indeed, the integration of infertility care services within UHC remains a challenge to be taken into account in the years to come” (Technical expert).

A majority of respondents felt that the priority of UHC in the context of service coverage was to provide adequate and quality healthcare services to the general public. There was, however, divergence on how this would be achieved in Morocco:

“…You know there are two medical cover schemes regulated through the law 65–00. About the UHC, currently, we have exceeded 68% of the rate of the covered population. The remainder is made up of health insurance for the self-employed, either in the formal or informal sector, which represents 31% of the total population but the health system should be able to provide health services with sufficient resources and quality of care.” (health insurance official).

Another point of discussion was highlighted by policy stakeholders about the absence of a central RH coordination committee. Though, its creation has been planned in the main lines of the national strategy of reproductive health, it is not yet implemented, therefore, weakening the implementation of an integrated approach toward universal access to SRH, especially in the current context of the organization of the Ministry of Health and the multitude of stakeholders in SRH matters. Also, the private sector is not integrated at the strategic level in sexual and reproductive health policy or programs. This fact is a structural problem of the Moroccan health system. The private sector lacks regulation and its activities are rarely documented.

This is why the Ministry of Health once again emphasizes that the future RH strategy must adopt an integrative approach within the UHC to all populations concerned by SRH:

“….at the strategic level, there is a fragmented RH programs. The integration of the components of SRH has benefits, it must be a priority. This integrative approach of the various services must be encouraged, including prioritizing reproductive health services to meet the emerging needs of the population.”(MOH official).

Thus, certain respondents mentioned the need to use new public health approaches to support the health system as self-care interventions for SRH which include new recommendation on self-screening with ovulation predictor kits (OPKs) for fertility management. Self-care interventions not only improve health outcomes, but can help to reduce poverty, promote gender equality and protect the most vulnerable populations contributing to UHC [26].

“to achieve UHC, we need to recognize the fact that communities must be more involved and informed, to prevent SRH diseases like infertility … On September 13, 2019, we launched "Self-Care" in Morocco as the first African and Arab country to ensure the integration of "Self-Care" in health policies, programs and practices at its national level toward UHC….” (NGO).

Although, the political context is very favorable and conducive for the prioritization of reproductive health services based on the needs of the population, gender sensitive and respecting human rights, the vertical organization of reproductive health programs, and the lack of an institutionalized coordination committee at the level of the Ministry of Health does not make it possible to take full advantage of these opportunities.

### Infertility characteristics

Stakeholders generally felt that there was a progress made in the monitoring of results and tracking of resources by the Ministry of Health. Since the 80 s, the periodic performance of the national population and family health survey “demographic and health surveys” enabled the Ministry to monitor the development of a certain number of indicators relating to fertility, mortality, maternal and child health, and family planning. However, there is no specified health indicator for infertility.

“…..Many departments are focusing on a core set of health indicators as part of the national plan. Progress is reported and reviewed on an annual basis. Performance SRH accountability done through monitoring of results via routine data, but some indicators as well as data from the private sector are mostly missing….” (MOH official).

The two key arguments against treatment of infertility in developing countries are overpopulation and limited resources [27]. Key informants representing the different stakeholder groups believe that primary health care is a complex process in which a variety of actors, structures and concerns need to be engaged, especially for infertility care services which remain very expensive. In addition, the difficulty of accessing infertility services exposes couples to childlessness and social stigma.

“…despite the medical technology advances of more than 30 years of IVF, only a small part of the population benefits from these new technologies. Time has come to give equitable access to effective and safe infertility care in the public health sector and reduce the infertility care services costs. Compared to countries in the region: Tunisia, which has only 11 million inhabitants, fully reimburses IVF and performs 10,000 cycles / year, compared to just 2,800 cycles in Morocco, which has a population of 35 million”. (Private sector representative).

Stakeholders who expressed concerns about the limited resources, proposed interventions based on cost minimization and standardization of services:

“…For me a minimum service to minimize costs and standardize services. This makes it possible to control the costs and the efficiency of services. Therefore, the minimum package is essential. You cannot let everyone do his or her own thing. It must be codified and it is very important…"(MOH official).

« …in my point of view, to insure availability and affordability of infertility care, the development plan must first affect cost reduction, this is an essential step to integrate infertility care services in to health benefit package. It’s a very complicated process». (Technical expert).

The stakeholders act according to a different logic; the public sector, the private sector, medicines and medical device companies, technical experts, civil society and NGOs. But it takes everyone's commitment to improve access to infertility care services:

“….now we start talking about Medically Assisted Procreation that must be integrated within the social security system. The cost is very high. So you have to define the conditions where it can be covered, at what age. We can propose it at least once. You need a systems approach. You have to tackle all the links. We cannot act on one link without the other" (Health insurance official).

### Outcome

Stakeholders considered that the health reforms in recent decades have been seriously represented by political achievements: developpement of the infertility regulation; establishement of the infertility action plan and the commitment of the national health insurance agency to include infertility medicines in the Health Benefit Package.

“Legal recognition, through law 14–47 on medically assisted procreation (MAP), of infertility as such allows infertile couples to overcome this social taboo which weighs with all its weight on the life of a couple in Morocco, in a society which considers infertility as a curse and which has for long wrongly attributed all responsibility to women”.(NGO).

A majority of respondents offered a few concrete solutions on how to better facilitate the role of the government in targeting and enrolling infertility care services to the SRH benefit package, highlighting the complexity of the infertility issue in Morocco and the need to translate political decisions into practical actions.

“…we are aware of the progress made, but infertile couples are asking me every day when will these actions be concretely felt; the law requires the implementing texts and the drugs are not yet effectively covered ….” (NGO).

However, some general suggestions offered by stakeholders ranged from the use of extended infertility care services coverage, costs reduction interventions, norms of infertility care services, continuity of regulation processes, innovative interventions for the population toward UHC like self-care interventions [28].

### Limitations

In our case study, we noted some limitations in the method used, as well as in the data collection process. Regarding the method used, the Shiffman and Smith framework does not take into consideration concerns about the implementation of the policy once it has been legislated; however, it guides us to highlight the problem analysis components that can be used to raise the profile of a condition to an actionable problem. Our study should be seen as a first exploration of this complex issue and of the neglect of certain health problems by decision-makers. Further research with innovative methods comparing health initiatives with varying levels of political support will be needed to identify the determining factors in setting the political priority for a country. Concerning the data collection; our study is based on qualitative data collected through interviews and focus group discussions; therefore, the quantitative measurements of the research items are not strongly reflected. The survey tools for the interviews highlighted SRH items, which induced limited responses to infertility subject. Furthermore, the survey analysis was based on interviewees’ perceptions leading to interview’s bias although it was minimized by the expertise of the sociologist who conducted the survey.

### Health policy implications and programs

The findings of this study showed that integrating infertility care services is influenced by several factors including: Power of actor and leadership, cohesion of different stakeholders, resources availability and requirements setting for prioritization in infertility within UHC.

Although efforts have been made in establishing the infertility regulation, more oriented actions need to be taken into consideration to insure the implementation of the preventive and therapeutic management of infertility care services. By exploring different factors of infertility services used in Morocco, this study revealed key items to be taken into consideration while implementing fertility service delivery with focus on women and couples needs as well as the capacities of health services provision and resources. This study reflected that policymakers did not systematically adopt human rights and gender-based principles while developing sexual and reproductive health programs. Therefore, it is recommended to ensure the convergence and alignment of all actors in translating the infertility regulation into measurable activities with defined human and financial resources, equitable fertility health coverage and the quality of infertility care responding to women and couples needs rights and dignity.

**Table:** 

Key message for policy makers:
1. Define infertility as a disease and set the infertility services as national priority to be integrated into the existing sexual and reproductive health package services
2. Translate the existing law 47.- 14 on Assisted Reproductive Technology into evidence based and high impact interventions at public and private health facilities
3. Organize a well defined path / flow for the couples suffering from infertility, in order for them to benefit from infertility prevention and management care services at primary health care services and referral hospitals
4. Secure required human and financial resources to ensure a delivery of fertility services within the health benefit package for better universal health coverage
5. Coordinate with all concerned stakeholders in establishing interconnected action plan for converged implementation and measurement
6. Document infertility care services practice and highlight the strengths and lessons learned to ensure better infertility universal health coverage in Morocco

## Conclusion

Despite a surge of interest in the world about infertility services by the global community, Morocco still failed to translate this issue into consistent political prioritization. Infertility care is an essential service within the SRH package to be provided in contribution to the UHC. Morocco has made progress in establishing the law for infertility, and has put infertility care as one of the components in the national reproductive health strategy since 2010. In addition, two infertility care units were created in tertiary hospitals to facilitate accessibility and affordability. However, with the joined collaboration and partnership of all concerned actors, more efforts need to be deployed for the translation of the existing regulation as well as the national ART action plan 2020–2030, therefore including infertility care within the integrated SRH package and benefiting from financial security. In order, for infertility care services to gain traction within the national RH political system, there is an urgent need for policy actors to use their technical and financial resources to create a more cohesive community of advocates and stakeholders, and to develop a clear problem definition of infertility care services and a public positioning of the matter. There is also a need to identify the leaders in the community whom are concerned by the problem and can act as policy entrepreneurs to facilitate the coupling of the problem of infertile couples with potential solutions when windows of opportunity are present, and therefore enable these couples to enjoy their maternity and paternity rights. 

## Data Availability

The datasets used and/or analysed during the current study are available from the corresponding author on reasonable request.

## Abbreviations

SRH: Sexual and Reproductive Health
UHC: Universal Health Coverage
IVFU: In Vitro Fertilization Units
MOH: Ministry of Health
ICPD: International Conference on Population and Development
RMH: Reproductive and Maternal Health
PHC: Primary Health Care
WHO: World Health Organization
MAP: Medically Assisted Procreation
ART: Assisted Reproductive Technology
CBRC: Cross-Border Reproductive Care
AMO: Mandatory Health Insurance
RAMED: Medical Assistance Scheme for the Economically Underprivileged
NGO: Non-Government Organization
HBP: Health Benefit Package
LMICs: Low- and Middle-Income Countries
OOP: Out-Of-Pocket

## Footnotes

1 Goal 3. Target 3.8: Achieve UHC, including financial risk protection, access to quality essential health-care services, and access to safe, effective, quality, and affordable essential medicines and vaccines for all.
